# Correlation Between Lower Lobe Dose Parameters and Radiation Pneumonitis in Definitive Radiation Therapy for Non‐Small Cell Lung Cancer

**DOI:** 10.1111/1759-7714.70369

**Published:** 2026-08-19

**Authors:** Sachiko Miura, Nobuhide Wakai, Shigeto Hontsu, Masahide Ota, Kaori Yamaki, Tetsuro Tamamoto, Masayuki Takeda, Fumiaki Isohashi

**Affiliations:** ^1^ Department of Radiation Oncology Nara Medical University Kashihara Nara Japan; ^2^ Department of Respiratory Medicine Nara Medical University Kashihara Nara Japan; ^3^ Department of Thoracic Oncology Kansai Medical University Medical Center Moriguchi Osaka Japan; ^4^ Department of Cancer Genomics and Medical Oncology Nara Medical University Kashihara Nara Japan; ^5^ Department of Strategic Promotion of Medical Information Cooperation Nara Medical University Kashihara Nara Japan

**Keywords:** carcinoma, non‐small‐cell lung, dose‐volume histogram, lobar‐specific parameters, radiation pneumonitis, radiotherapy

## Abstract

**Background:**

This study examined whether lobar‐specific dose‐volume histogram (DVH) parameters add predictive information for radiation pneumonitis (RP) beyond conventional whole‐lung parameters in patients with non‐small cell lung cancer (NSCLC) undergoing definitive radiotherapy.

**Methods:**

We retrospectively analyzed 145 NSCLC patients treated between 2018 and 2023. Conventional whole‐lung parameters, including V5%, V20%, and mean lung dose (MLD), lobar‐specific parameters, including Low V20% (percentage of the bilateral lower lobes receiving ≥ 20 Gy), and clinical parameters were analyzed. The predictive performance of each parameter for Grade ≥ 2 RP was evaluated using receiver operating characteristic curve analysis. Associations with Grade ≥ 2 RP were assessed by Gray test and Fine‐Gray competing risks regression.

**Results:**

Grade ≥ 2 RP had an incidence of 41% (60/145) and showed significant associations with V20% (*p* = 0.005), MLD (*p* = 0.025), Low V5% (*p* = 0.006), and Low V20% (*p* = 0.003). In multivariate analysis, a high Brinkman Index (BI) (HR = 2.10, 95% CI: 1.27–3.48, *p* = 0.004) and high Low V20% (HR = 2.31, 95% CI: 1.12–4.74, *p* = 0.023) were independent predictors for Grade ≥ 2 RP. The correlation between V20% and Low V20% was moderate (*r* = 0.505), with minimal multicollinearity (variance inflation factor 1.34).

**Conclusion:**

Low V20% was independently associated with Grade ≥ 2 RP and may provide complementary information to conventional whole‐lung DVH parameters and BI. This simple lobar‐specific parameter may help refine pretreatment RP risk stratification, although external validation is required before its incorporation into individualized planning constraints.

## Graphical abstract

Among 145 patients with NSCLC receiving definitive thoracic RT, Low V20% (percentage of bilateral lower lobes receiving ≥ 20 Gy) was significantly associated with Grade ≥ 2 radiation pneumonitis. High Low V20% independently predicted Grade ≥ 2 radiation pneumonitis (HR 2.31, 95% CI 1.12–4.74), suggesting added value for pretreatment risk stratification.

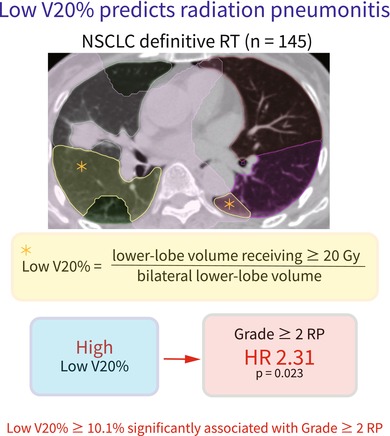

## 1 Introduction

Definitive thoracic radiation therapy (RT) delivered with or without concurrent chemotherapy is an important curative‐intent treatment for patients with non‐small cell lung cancer (NSCLC). Concurrent chemoradiotherapy (cCRT) is widely established as definitive treatment for locally advanced NSCLC, whereas RT alone remains clinically important for elderly patients and those with comorbidities [1]. In both settings, radiation pneumonitis (RP) is a clinically relevant adverse event that may impair quality of life, require corticosteroid therapy or limit subsequent systemic treatment. Recent advances have led to incorporation of maintenance therapy with immune checkpoint inhibitors (ICIs) or tyrosine kinase inhibitors following cCRT, which has improved outcomes [2, 3]. For instance, the PACIFIC study showed that durvalumab maintenance therapy improved overall survival and progression‐free survival (PFS) [2]. Similarly, osimertinib maintenance therapy has been shown to significantly improve PFS in locally advanced NSCLC with EGFR mutations [3]. While these maintenance therapies have become standard treatments, their introduction has emphasized the clinical significance of RP, a well‐known adverse event following definitive RT. Because onset of RP often overlaps with the initiation period for maintenance therapy, development of RP may be a potential clinical risk that could delay or preclude initiation of maintenance therapy [4, 5].

For decades, whole‐lung dose‐volume histogram (DVH) parameters, such as the percentage of volume receiving ≥ 20 Gy (V20%) and the mean lung dose (MLD), have been used as robust predictors for RP [6, 7, 8]. Recently, the utility of the percentage of volume receiving ≥ 5 Gy (V5%) has been reported, though findings remain inconsistent [9, 10]. Given the growing importance of accurate prediction and evaluation of RP, there is a need for more precise predictors than these conventional parameters. Therefore, we focused on lobar‐specific respiratory function, particularly that of the lower lobes, with the aim of identifying novel DVH parameters. Several reports have indicated that the lower lobes play a more significant functional role than the upper lobes, and that irradiation of the lower lobes is associated with a higher risk of RP [11, 12, 13, 14, 15, 16, 17, 18]. Based on the hypothesis that dose exposure to the more functional lower lobes is a critical determinant of RP risk, we examined if lobar‐specific DVH parameters could predict Grade ≥ 2 RP in patients with NSCLC undergoing definitive RT.

## 2 Methods

### 2.1 Study Design

This retrospective study was approved by the Nara Medical University Institutional Ethics Committee and was performed in accordance with the Declaration of Helsinki. Informed consent was waived.

### 2.2 Patients

**FIGURE 1:**
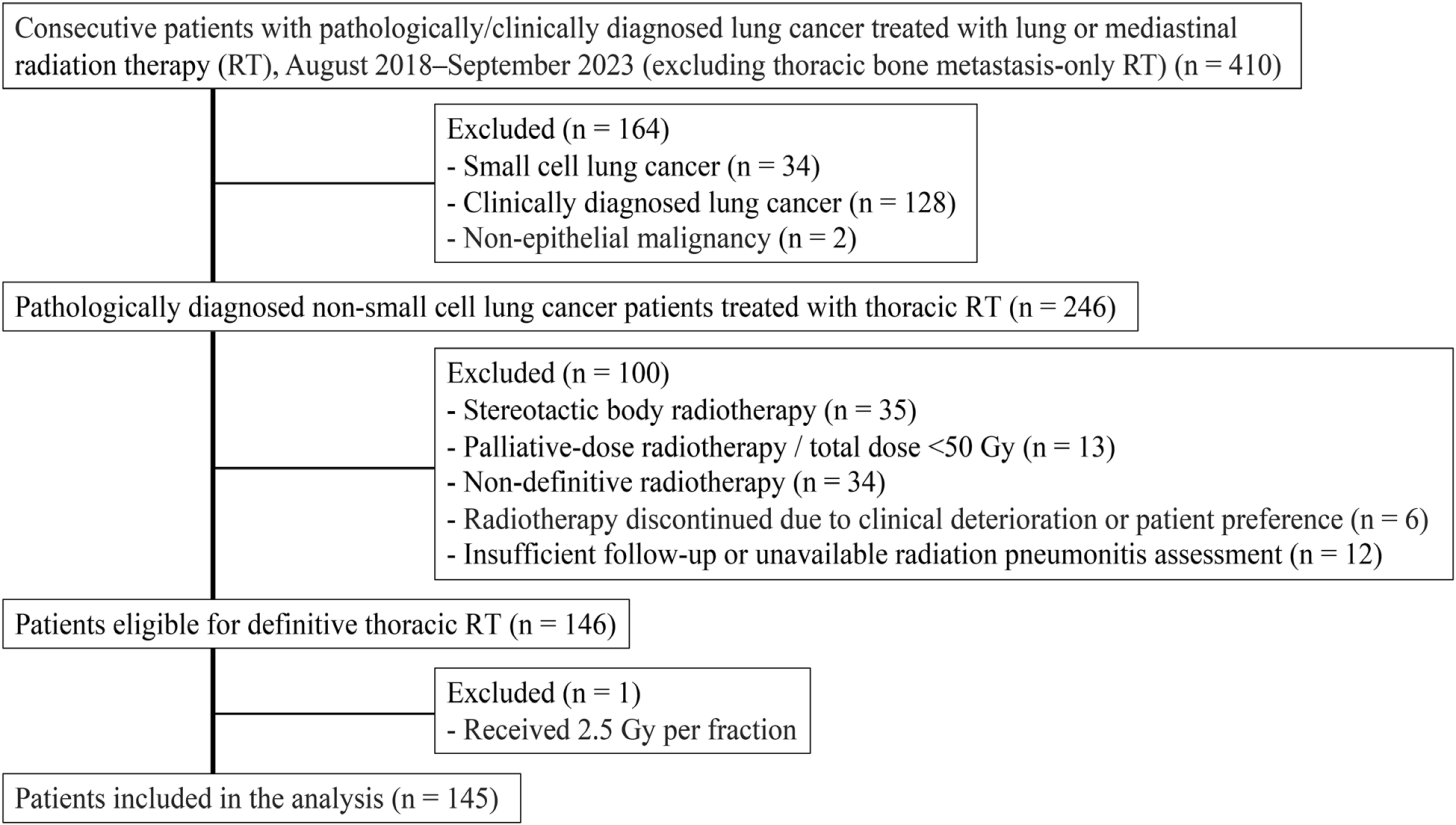
Patient flow diagram.

Patient selection is summarized in Figure 1. We retrospectively screened consecutive patients with pathologically or clinically diagnosed lung cancer who underwent RT to the lung or mediastinum at our center between August 2018 and September 2023, excluding those treated only for thoracic bone metastases. Definitive thoracic RT was defined as conventionally fractionated, curative‐intent thoracic RT with a prescribed total dose ≥ 50 Gy. Stereotactic body radiotherapy, palliative‐dose RT with a total dose < 50 Gy, nondefinitive RT, cases in which RT was discontinued, cases with insufficient follow‐up or unavailable RP assessment, and patients treated with fraction sizes other than 2.0 Gy were excluded, as shown in Figure 1. Finally, 145 patients with pathologically diagnosed NSCLC were included in the analysis.

### 2.3 Radiation Therapy

For treatment planning, patients were immobilized in a supine position and underwent a planning CT scan (Aquilion LB, Toshiba Medical Systems, Tokyo, Japan) using a free‐breathing protocol. Treatment planning was performed using Eclipse software (Eclipse v.13.5, Varian, California, USA). Dose calculations were performed using the AAA algorithm. All RT fractions were delivered under free‐breathing conditions. Treatment techniques included three‐dimensional conformal radiation therapy (3D‐CRT), intensity‐modulated radiation therapy (IMRT), and a combination of both. Based on a comprehensive assessment of tumor size and lung function, either elective nodal irradiation (ENI) or involved‐field radiation therapy (IFRT) was performed.

The gross tumor volume (GTV) included the primary tumor and involved lymph nodes. The nodal GTV (GTVn) was defined as ^18^F‐FDG PET‐positive lymph nodes. Organs at risk, including the whole lung, esophagus, spinal cord, and heart, were automatically contoured during treatment planning. For ENI treatment planning, the clinical target volume of nodes (CTVn) included the mediastinal lymph node stations containing GTVn, adjacent stations, and in some cases, the next adjacent stations, as contoured by a radiation oncologist. For IFRT treatment planning, CTVn was defined as GTVn plus a 5‐mm margin in all directions. The planning target volume was determined from the clinical target volume using an automatic margining tool that added a standard margin of 5 mm.

### 2.4 Dosimetry Evaluations

**FIGURE 2:**
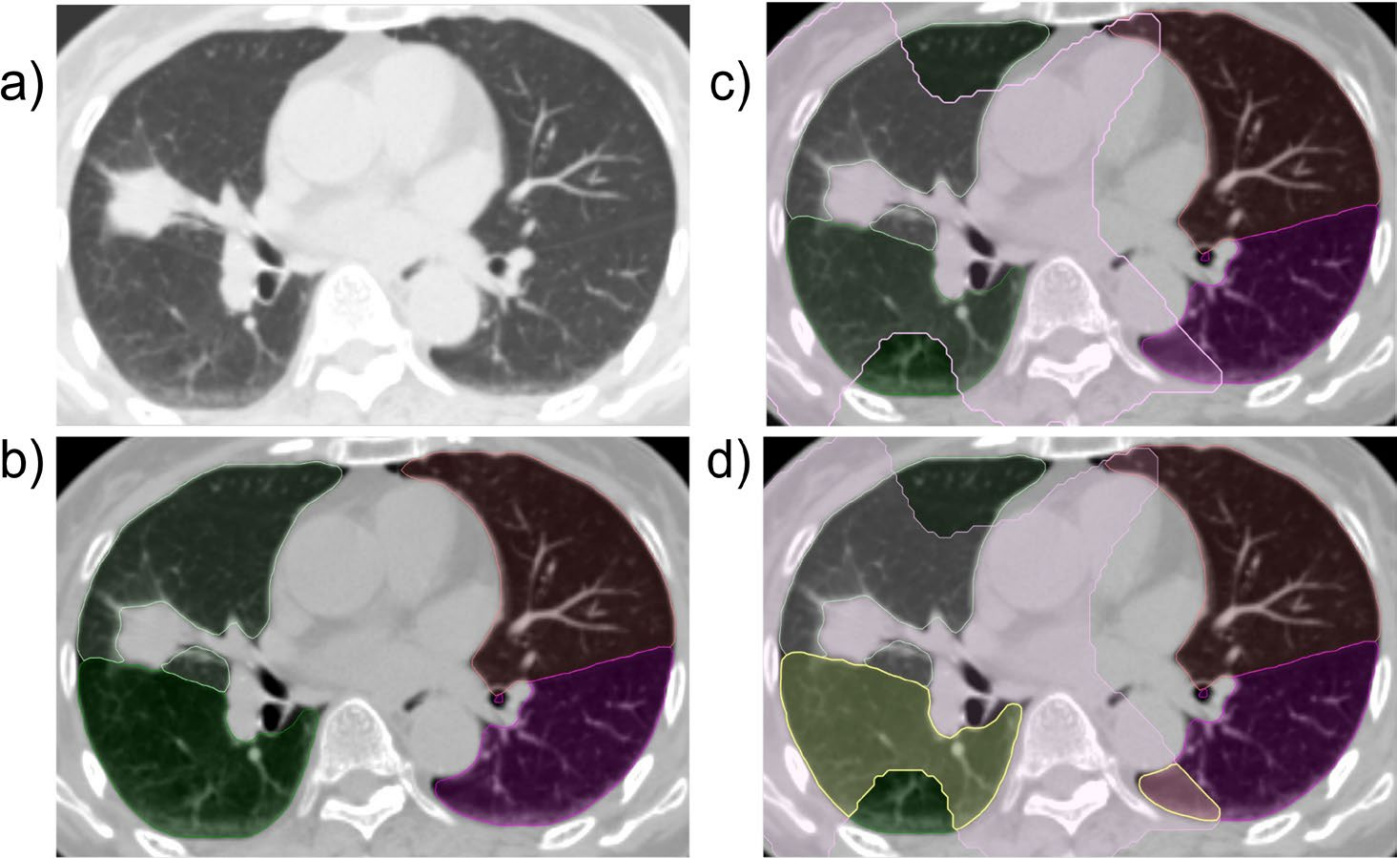
(a) A case of right middle lobe lung cancer. Axial image of lung window settings on the treatment planning contour screen. Bilateral major fissures are visible. (b) Four regions were separately contoured: Right upper and middle lobes, right lower lobe, left upper lobe, and left lower lobe. The GTV was excluded from the lung contour. (c) The light pink region is the area receiving ≥ 20 Gy for the total lung (V20%). (d) The yellow region is the area receiving ≥ 20 Gy in the bilateral lower lobes.

Standard whole‐lung parameters were calculated prior to treatment, including V5%, V20%, and MLD. The whole lung was defined as the volume of both lungs minus the GTV. Lobar‐specific parameters were calculated retrospectively. The lung window settings on the treatment planning contour screen were manually adjusted to identify the bilateral major fissures. The right upper and middle lobes, right lower lobe, left upper lobe, and left lower lobe were separately contoured by two experienced radiation oncologists. Based on anatomical and functional similarities, the right upper/middle lobes and left upper lobe were combined into a composite “Upper lobes” region, while the bilateral lower lobes formed the “Lower lobes” region [18, 19, 20]. For these regions, the absolute volumes receiving ≥ 5 Gy (Up V5 cc; Low V5 cc) and ≥ 20 Gy (Up V20 cc; Low V20 cc) were defined. The corresponding percentage volumes were then calculated by dividing each absolute volume by the total volume of the upper or lower lobe: Up V5%, Low V5%, Up V20%, and Low V20% (Figure 2). RP was evaluated using Common Terminology Criteria for Adverse Events version 5.0. The grade of RP was recorded as the highest grade observed during follow‐up. For patients with Grade ≥ 2 RP, the onset date was defined as the date on which Grade 2 RP was first documented, while patients with Grade ≤ 1 RP were censored at the date of final follow‐up.

### 2.5 Evaluation of Pretreatment Pulmonary Fibrosis Score

Pretreatment CT images were re‐reviewed to evaluate pulmonary fibrosis using the pulmonary fibrosis score described by Tsujino et al. [21].

### 2.6 Follow‐Up

Patients were followed up monthly, and CT was performed every 3 months after completion of RT. All CT images were independently reviewed by certified thoracic radiologists to ensure accurate identification of even subtle imaging changes corresponding to Grade 1 RP.

### 2.7 Statistical Analysis

Receiver operating characteristic (ROC) curves were constructed, and the area under the curve (AUC) was calculated for each lung parameter to evaluate its predictive capability for Grade ≥ 2 RP. Univariate analyses of the relationship between clinical/dosimetric parameters and the incidence of Grade ≥ 2 RP were conducted using a Gray test, with stratification based on optimal cut‐off values determined by ROC analysis. Death before development of Grade ≥ 2 RP was treated as a competing event. The Holm method was used to adjust *p*‐values for multiple comparisons across all tested dosimetric parameters. Multivariate analysis was performed using a Fine‐Gray proportional hazards model to identify independent risk factors. Variables for inclusion (e.g., age, Brinkman Index (BI), V5%, V20%, MLD, and Low V20% or Low V20 cc) were selected based on clinical relevance and univariate results. A Pearson correlation coefficient and variance inflation factor (VIF) were calculated to assess the relationship and potential multicollinearity between V20% and Low V20%. Since durvalumab initiation occurred after cCRT and could be influenced by early onset of RP or clinical judgment, durvalumab was not treated as a baseline covariate. Because durvalumab maintenance therapy is generally initiated within 42 days after completion of cCRT [2], we performed a 42‐day landmark analysis among cCRT patients after excluding RT‐alone cases and those that developed Grade ≥ 2 RP within 42 days after completion of RT. Early post‐cCRT CT images before the 42‐day landmark were reviewed to assess the presence of radiographic RP. All statistical analyses were performed using EZR software (Saitama Medical Center, Jichi Medical University, Saitama, Japan). A *p*‐value < 0.05 was considered statistically significant.

## 3 Results

The patient characteristics are shown in Table 1. Smoking history was present in 125 patients (86%), with a median BI of 920 (interquartile range (IQR) 400–1200). The tumor locations were 92 cases (63.4%) in the upper and middle lobe, 41 (28.3%) in the lower lobe, and 12 (8.3%) in the mediastinum. There was no documented clinical diagnosis of interstitial pneumonia. However, re‐review of pretreatment CT images showed pulmonary fibrosis scores of 0, 1, 2, and 3 in 86, 37, 16, and 6 patients, respectively, indicating that patients with mild or asymptomatic interstitial abnormalities on CT were included in the analysis. Grade ≥ 2 RP occurred in 35/86 (41%), 14/37 (38%), 7/16 (44%), and 4/6 (67%) patients, respectively.

**TABLE 1:** Patient and treatment characteristics (*n* = 145).

Item	Median	*n*	%
Age (range)	72 (41–90)		
Gender			
Male		113	77.9
Female		32	22.1
Smoking history			
Ever smoker		125	86.2
Brinkman Index (IQR)	920 (400–1200)		
Never smoker		20	13.8
Brinkman Index (IQR)	0		
ECOG‐PS			
0		75	51.7
1		52	35.9
2		18	12.4
Stage (UICC 8th edition)			
I		3	2.1
II		26	17.9
III		102	70.3
IVA		2	1.4
Postsurgery		12	8.3
Pathology			
Adenocarcinoma		58	40.0
Squamous cell carcinoma		57	39.3
Others		30	20.7
Tumor location			
Upper or middle		92	63.4
Lower		41	28.3
Mediastinum		12	8.3
Lung function (IQR)			
% Vital capacity	88.6 (78.4–103.2)		
% FEV1.0	75.5 (61.9–88.6)		
Pulmonary fibrosis score			
0		86	59.3
1		37	25.5
2		16	11.0
3		6	4.1
4 or 5		0	0
Chemotherapy			
Yes		86	59.3
No		59	40.7
Chemotherapy regimen			
Carboplatin + paclitaxel		8	9.3
Carboplatin + nab‐paclitaxel		51	59.3
Cisplatin + docetaxel		26	30.2
Tegafur/uracil (UFT)		1	1.2
Durvalumab			
Yes		44	30.3
No		101	69.7
Planning target volume (cc) (IQR)	378.9 (269.9–573.9)		
Dose (Gy) (IQR)	66 (60–66)		
Number of fractions (IQR)	33 (30–33)		
Radiation method			
3D‐CRT		77	53.1
Combined		32	22.1
IMRT		36	24.8
Radiation field			
Elective nodal irradiation		125	86.2
Involved field radiotherapy		20	13.8

Abbreviations: 3D‐CRT, three‐dimensional conformal radiation therapy; ECOG‐PS, Eastern Cooperative Oncology Group performance status; FEV1.0, forced expiratory volume in 1 s; IMRT, intensity‐modulated radiation therapy; IQR, interquartile range; UICC, Union for International Cancer Control.

A total of 86 patients received cCRT and 59 received RT alone, of whom 55 (93.2%) had Stage II–III disease. cCRT was not used for patients who were elderly and/or had a poor performance status (*n* = 33), those with a comorbidity or other conditions (*n* = 22), and due to patient preference (*n* = 4). Among the 86 cCRT cases, 85 received a taxane‐containing regimen and one received tegafur‐uracil. The two stage IV patients had oligometastatic disease and were treated with definitive thoracic RT. No patient received ICIs concurrently with thoracic RT. The median follow‐up period was 19.0 months (IQR 9.0–37.1 months). Five patients had no RP, 80 had Grade 1, 54 had Grade 2, and 6 had Grade 3; thus, Grade ≥ 2 RP occurred in 60 patients (41%). There was no Grade 4 or 5 RP. Of the 54 patients with Grade 2 RP, 3 required steroid pulse therapy and were considered to be clinically severe Grade 2 cases.

Results for each lung parameter are shown in Table 2. The median Low V20% was 13.9 (IQR 8.3–20.8; range, 0–53.0). Seven patients had Low V20% = 0 and were retained in the primary analysis. Table 3 shows the results of ROC and univariate analyses. Among clinical parameters, BI was significantly associated with Grade ≥ 2 RP (*p* = 0.013). Durvalumab initiation showed an inverse association with Grade ≥ 2 RP (*p* = 0.003). However, durvalumab was considered to be a postradiotherapy treatment variable; therefore, its association with Grade ≥ 2 RP was further evaluated using landmark analysis. After excluding the 59 RT alone cases and 13 cCRT cases with Grade ≥ 2 RP within 42 days, 73 patients remained. Grade ≥ 2 RP occurred in 10/42 patients who started durvalumab within 42 days and 12/31 patients who did not, with no significant difference (Gray test *p* = 0.144) (Table S1). Early post‐cCRT CT findings of radiographic RP before the 42‐day landmark were absent in 58 patients, present in 13, and not evaluable in 2. Subsequent Grade ≥ 2 RP occurred in 24.1%, 53.8%, and 50.0% of these patients, respectively (Table S2).

**TABLE 2:** Lung dosimetric parameters.

Lung parameters	Median (IQR)	Variables
V5%	39.3 (31.3–49.5)	
V20%	22.5 (18.6–26.8)	
Mean lung dose (Gy)	13.8 (11.3–16.1)	
Total lung volume (cc)	3146 (2446–3886)	
Volume of both lower lobes (cc)	1299 (940–1645)	
Low V5%	24.5 (15.8–36.5)	
Up V5%	46.8 (37.8–62.3)	
Low V20%		
Range		0–53.0
Number of patients with Low V20% = 0		7
Tumor location: upper/middle lobes	12.8 (8.5–19.3)	
Tumor location: lower lobe	18.9 (9.3–27.9)	
Total	13.9 (8.3–20.8)	
Up V20%	26.8 (21.4–32.5)	
Low V5 cc	326.3 (188.2–471.4)	
Up V5 cc	878.2 (646.7–1129.7)	
Low V20 cc		
Tumor location: upper/middle lobes	175.6 (105.6–271.8)	
Tumor location: lower lobe	269.1 (107.7–358.1)	
Total	183.8 (102.5–300.9)	
Up V20 cc	478.1 (343.1–616.5)	

Abbreviation: IQR, interquartile range.

**TABLE 3:** Univariate analyses of parameters related to Grade ≥ 2 radiation pneumonitis.

Parameter	ROC analysis		Univariate analysis	
	AUC	Threshold	Statistic (*χ* ^2^)	*p*
Clinical parameters				
Brinkman Index	0.581	1060.0	6.177	0.013
ECOG‐PS				
0 versus 1–2	NA	NA	2.177	0.140
Pulmonary fibrosis score				
0 versus 1–3	NA	NA	0.147	0.701
0–1 versus 2–3	NA	NA	0.718	0.397
cCRT versus RT alone	NA	NA	0.290	0.590
With versus without Durvalumab	NA	NA	9.018	0.003
IMRT versus 3D‐CRT	NA	NA	1.417	0.234
ENI versus IFRT	NA	NA	1.054	0.305
Dosimetric parameters (%)				
V5%	0.544	35.7	3.191	0.074
V20%	0.608	22.5	7.801	0.005
Mean lung dose (Gy)	0.598	15.4	4.999	0.025
Low V5%	0.597	28.5	7.688	0.006
Up V5%	0.510	58.6	0.596	0.440
Low V20%				
Tumor location: upper/middle lobes	0.657	10.1	9.033	0.003
Tumor location: lower lobe	0.625	27.2	3.771	0.052
Total	0.618	10.1	8.854	0.003
Up V20%	0.523	28.9	1.388	0.239
Dosimetric parameters (absolute)				
Low V5 cc	0.571	370.9	3.825	0.051
Up V5 cc	0.522	668.8	0.597	0.440
Low V20 cc	0.612	191.1	10.564	0.001
Up V20 cc	0.517	556.4	2.001	0.157

*Note:* Threshold values represent the optimal cutoff values derived from ROC analysis.

Abbreviations: 3D‐CRT, three‐dimensional conformal radiation therapy; AUC, area under the curve; cCRT, concurrent chemoradiotherapy; ENI, elective nodal irradiation; IFRT, involved field radiotherapy; IMRT, intensity‐modulated radiotherapy; ROC, receiver operating characteristic; RT, radiotherapy.

Among the dosimetric parameters in Table 3, there were significant associations with V20% (*p* = 0.005), MLD (*p* = 0.025), Low V5% (*p* = 0.006), Low V20% (overall, *p* = 0.003; upper/middle lobe tumors, *p* = 0.003), and Low V20 cc (*p* = 0.001). Low V20% was significantly associated with Grade ≥ 2 RP overall and for upper/middle‐lobe tumors, but did not reach significance for lower‐lobe tumors (AUC 0.625, *p* = 0.052). A sensitivity analysis excluding patients with Low V20% = 0 yielded similar results: Low V20% remained significant (AUC 0.616, *p* = 0.004), as did Low V20 cc (AUC 0.609, *p* = 0.002) (Table S3).

In multivariate analyses incorporating six factors: age, BI (< 1060 vs. ≥ 1060), V5% (< 35.7 vs. ≥ 35.7), V20% (< 22.5 vs. ≥ 22.5), MLD (< 15.4 vs. ≥ 15.4), and Low V20% (< 10.1 vs. ≥ 10.1) (thresholds from ROC analysis), there were significant associations with Grade ≥ 2 RP for BI (HR = 2.101, 95% CI: 1.27–3.48, *p* = 0.004) and Low V20% (HR = 2.31, 95% CI: 1.12–4.74, *p* = 0.023) (Table 4). The Pearson correlation coefficient between V20% and Low V20% was moderate (*r* = 0.505), and VIF was 1.34, indicating minimal multicollinearity. A separate model substituting Low V20% with Low V20 cc (< 191.1 vs. ≥ 191.1) also showed a strong association with Grade ≥ 2 RP (BI: HR = 1.98, 95% CI: 1.20–3.26, *p* = 0.007; Low V20 cc: HR = 2.31, 95% CI: 1.33–4.02, *p* = 0.003) (Table S4).

**TABLE 4:** Multivariate analyses using the Fine‐Gray method for Grade ≥ 2 radiation pneumonitis.

Variables	HR	95% CI	*p*
Age	1.008	0.981–1.036	0.560
Brinkman Index (< 1060 vs. ≥ 1060)	2.101	1.269–3.478	0.004
V5% (< 35.7 vs. ≥ 35.7)	0.964	0.465–2.001	0.920
V20% (< 22.5 vs. ≥ 22.5)	1.532	0.597–3.930	0.370
Mean lung dose (Gy) (< 15.4 vs. ≥ 15.4)	1.074	0.504–2.287	0.850
Low V20% (< 10.1 vs. ≥ 10.1)	2.309	1.124–4.741	0.023

Abbreviations: CI, confidence interval; HR, hazard ratio.

In cCRT cases, Low V20% had the highest AUC among all dosimetric parameters (AUC 0.675, *p* = 0.002). Low V20 cc was also strongly associated with Grade ≥ 2 RP (AUC 0.668, *p* = 0.001) (Table S5). Additional laterality‐specific lobar analyses gave broadly similar trends for V20% and V20 cc. ROC direction was interpreted according to the side of the threshold associated with higher Grade ≥ 2 RP risk. For right‐sided parameters, values above the threshold, and for left‐sided parameters, values below the threshold were associated with a higher risk of Grade ≥ 2 RP. Significant associations with Grade ≥ 2 RP were observed for all right‐sided parameters and for the left upper lobe, whereas left lower lobe parameters were not significant (Table S6).

**FIGURE 3:**
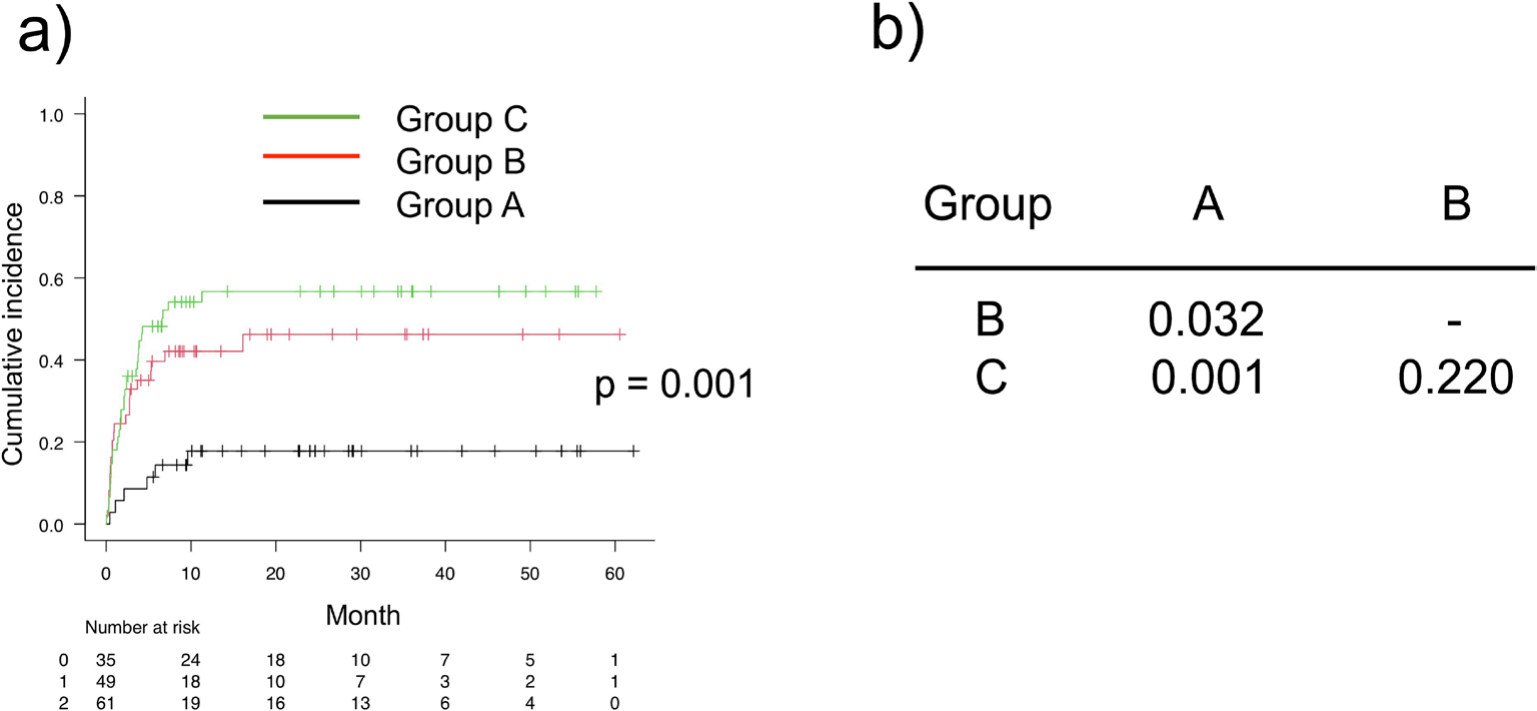
Risk stratification for Grade ≥ 2 radiation pneumonitis (RP) based on V20% and Low V20%. Patients were stratified into three groups based on receiver operating characteristic (ROC)‐derived cut‐off values for V20% and Low V20%: Group A (low V20% and low Low V20%), Group B (discordant, one high/one low), and Group C (high V20% and high Low V20%). (a) Cumulative incidence curves were generated using the Gray test. (b) *p*‐values were adjusted for multiple comparisons using the Holm method.

V20% and Low V20% had a moderate correlation (*r* = 0.505), with minimal multicollinearity (VIF 1.34). To further evaluate the associations, patients were stratified into three groups based on ROC‐derived cut‐off values for V20% and Low V20%: Group A: Low V20% and low Low V20% (both below cut‐off), Group B: discordant (one parameter high, one low), and Group C: High V20% and high Low V20% (both above cut‐off). Holm analysis identified significant differences in the incidence of Grade ≥ 2 RP between Groups A and B, and between Groups A and C (Figure 3).

## 4 Discussion

In this study, lower‐lobe dose parameters, particularly Low V20%, were significantly associated with Grade ≥ 2 RP in patients with NSCLC undergoing definitive thoracic RT. Conventional whole‐lung DVH parameters such as V20% and MLD remain important and clinically established predictors of RP; however, they treat the lung as a relatively uniform organ and may not fully capture regional differences in pulmonary function or radiosensitivity. Low V20% is a simple lobar‐specific parameter that reflects dose exposure to the bilateral lower lobes and may provide complementary information to conventional whole‐lung DVH parameters.

Our findings suggest that Low V20% captures a dosimetric concept distinct from conventional V20%. The correlation between V20% and Low V20% was moderate, and VIF was low, indicating minimal multicollinearity. Thus, Low V20% does not merely duplicate whole‐lung V20%, but rather reflects the regional distribution of dose in functionally important lower‐lobe lung tissue. Low V20 cc also showed a similar association with Grade ≥ 2 RP; however, percentage‐based parameters are generally more robust against interpatient differences in lung volume and respiratory phase. For this reason, Low V20% was selected as the primary lower‐lobe parameter in the multivariable analysis.

The increased radiosensitivity of the lower lobes may be explained by their distinct physiology. Pulmonary perfusion is gravity‐dependent, resulting in greater perfusion in the lower lobes. These lobes are also more easily expanded by diaphragmatic movement, leading to greater ventilation volume. Therefore, the lower lobes have abundant perfusion and ventilation, resulting in a higher oxygen supply. Given the known involvement of oxidative stress and inflammatory cytokines in RP [22, 23, 24], these processes may be intensified in the lower lobes due to their high perfusion, ventilation, and oxygen supply, potentially leading to increased susceptibility to radiation‐induced inflammation. Furthermore, gravity may promote accumulation of inflammatory substances in the lower lobes, prolonging and exacerbating inflammation [25]. Preclinical studies in rodents also support regional variations in radiosensitivity [26], suggesting a higher density of target cells in the lung base [27] and increased out‐of‐field effects with lower lung irradiation [28].

The clinical relevance of Low V20% should be interpreted in the context of modern combined‐modality treatment. With the introduction of maintenance therapies after cCRT, even Grade 2 RP may become clinically important if it delays or precludes subsequent systemic treatment. RT alone also remains clinically important, particularly for elderly patients and those with comorbidities. Modern RT techniques, including IMRT, also have greater flexibility in shaping dose distributions. Therefore, a simple regional parameter such as Low V20% may be useful for pretreatment risk stratification and for identifying patients in whom careful lower‐lobe dose evaluation is warranted. However, our results do not establish Low V20% as a universal dose constraint, since Low V20% was not associated with Grade ≥ 2 RP in patients with lower‐lobe tumors. In this subgroup, an increased Low V20% may largely reflect unavoidable irradiation of the lower lobes due to tumor location, rather than a planning factor that can be readily modified.

Several additional analyses support, but do not definitively prove, the robustness of the main finding. Patients with Low V20% = 0 were retained in the primary analysis because this value represents the true absence of lower‐lobe volume receiving ≥ 20 Gy. Sensitivity analysis excluding these patients yielded similar results. In the cCRT subgroup, Low V20% also showed a similar or slightly stronger association with Grade ≥ 2 RP, supporting the applicability of this parameter in patients receiving combined‐modality therapy. The laterality‐based analysis was exploratory and should be interpreted cautiously, as the observed associations may reflect cohort‐specific factors such as tumor laterality, nodal involvement, field arrangement, lung‐volume differences, and dose distribution, rather than intrinsic biological differences between the right and left lungs.

Among clinical factors, a high BI was independently associated with Grade ≥ 2 RP. Previous studies have reported inconsistent associations between smoking‐related variables and RP, and this finding should therefore be interpreted cautiously [29, 30]. However, our BI finding may not be entirely inconsistent with those reports. BI is a cumulative quantitative index of smoking burden and should be distinguished from categorical smoking status. A high BI may reflect long‐term smoking‐related lung injury, emphysema, airway inflammation, vascular damage, and reduced pulmonary reserve, all of which could lower the threshold for symptomatic radiation‐induced lung injury. Clinically significant interstitial pneumonia and CT‐based interstitial abnormalities are also important risk factors for RP. No patient in our cohort had a documented clinical diagnosis of interstitial pneumonia, but mild or asymptomatic interstitial abnormalities were identified on re‐review of pretreatment CT images. Definitive interpretation is limited because only a few patients had higher fibrosis scores, and clinically significant lung disease may have been underrepresented in the cohort.

The inverse association between durvalumab use and RP in the unadjusted analysis should not be interpreted as a protective effect of durvalumab. Durvalumab initiation is a postradiotherapy decision influenced by patient fitness, early symptoms, radiographic findings, and physician judgment. The 42‐day landmark analysis attenuated this apparent association, supporting the possibility of clinical selection, rather than causality. Grade 2 RP is also clinically heterogeneous, and some Grade 2 cases in the cohort required steroid pulse therapy. Therefore, future studies should evaluate more clinically specific endpoints, such as prolonged corticosteroid use, steroid pulse therapy, hospitalization, oxygen requirement, and interruption or discontinuation of maintenance therapy.

This study has several limitations. First, it was a single‐center retrospective study, and selection bias and unmeasured confounders may remain despite the inclusion of consecutive patients. Second, treatment modality was not randomized; patients treated with cCRT and RT alone differed in clinical background, pulmonary reserve, and eligibility for maintenance therapy. Third, almost all cCRT cases received taxane‐containing regimens, so the independent effect of the chemotherapy regimen could not be evaluated. Fourth, due to the retrospective design, the independent contribution of dosimetric risk could not be determined precisely, as the observed association may have been influenced by clinical decision‐making regarding durvalumab initiation, even with landmark analysis. Fifth, RP grading may have been influenced by baseline clinical status, symptom subjectivity, physician judgment, and documentation of steroid use. Finally, the subgroup, sensitivity, and laterality analyses were exploratory and require external validation.

In conclusion, Low V20% was significantly associated with Grade ≥ 2 RP in patients undergoing definitive thoracic RT for NSCLC. This simple lobar‐specific parameter may provide complementary information to conventional whole‐lung DVH parameters and may help refine pretreatment risk stratification. Further validation in independent multicenter cohorts is required before Low V20% can be incorporated into individualized planning constraints.

## Author Contributions

**Masahide Ota:** investigation, writing – review and editing. **Masayuki Takeda:** writing – review and editing. **Shigeto Hontsu:** investigation, writing – review and editing. **Tetsuro Tamamoto:** writing – review and editing. **Nobuhide Wakai:** investigation, writing – review and editing. **Sachiko Miura:** conceptualization, methodology, formal analysis, writing – original draft, data curation. **Kaori Yamaki:** investigation, writing – review and editing. **Fumiaki Isohashi:** writing – review and editing, supervision.

## Funding

The authors have nothing to report.

## Ethics Statement

This study was approved by the Nara Medical University Institutional Ethics Committee (approval No. 1952), and was performed in accordance with the Declaration of Helsinki.

## Consent

The requirement for informed consent was waived by the Institutional Ethics Committee due to the retrospective study.

## Conflicts of Interest

The authors declare no conflicts of interest.

## Supporting information


**Table S1:** 42‐day landmark analysis (*n* = 73).
**Table S2:** Association between early radiographic radiation pneumonitis (RP) findings after cCRT and subsequent Grade ≥ 2 RP in the landmark cohort (*n* = 73).
**Table S3:** Sensitivity analysis excluding patients with Low V20% = 0 (*n* = 138).
**Table S4:** Multivariate analyses using the Fine‐Gray method for Grade ≥ 2 radiation pneumonitis.
**Table S5:** cCRT‐only analysis (*n* = 86).
**Table S6:** Laterality‐based analysis (*n* = 145).

## Data Availability

The datasets generated and/or analyzed during the current study are not publicly available due to patient privacy and institutional regulations, but are available from the corresponding author on reasonable request.
