# Supplementary material for: Correlation Between Lower Lobe Dose Parameters and Radiation Pneumonitis in Definitive Radiation Therapy for Non‐Small Cell Lung Cancer
Source: Thorac Cancer. 2026 Aug 19;17(16):e70369. doi: 10.1111/1759-7714.70369 (PMC13487692; doi:10.1111/1759-7714.70369)
Supplement: Supplementary file 1 [file TCA-17-e70369-s001.docx]

Supplementary Table 1. 42-day landmark analysis (n=73)

|  | Statistic (χ^2^) | | *p-value* |
| --- | --- | --- | --- |
| Durvalumab within 42 days vs. no durvalumab within 42 days | 2.130 | 0.144 | |

Supplementary Table 2. Association between early radiographic radiation pneumonitis (RP) findings after cCRT and subsequent Grade ≥ 2 RP in the landmark cohort (n=73)

| Early radiographic RP findings | n | Grade ≥ 2 RP | | % |
| --- | --- | --- | --- | --- |
| No  Yes  Not evaluable | 58  13  2 | | 14  7  1 | 24.1  53.8  50.0 |

Supplementary Table 3. Sensitivity analysis excluding patients with Low V20% = 0 (n=138)

| **Parameter** | **ROC analysis** | |  | **Univariate analysis** | | |  |
| --- | --- | --- | --- | --- | --- | --- | --- |
|  | **AUC** | **Threshold** |  | **Statistic (χ^2^)** | | ***p-value*** | |
| **Dosimetric parameters (%)**  V5% | 0.470 | 57.5 |  | 0.163 | 0.686 | |  |
| V20% | 0.595 | 22.5 |  | 5.812 | 0.016 | |  |
| Mean lung dose (Gy)  Low V5%  Up V5%  Low V20%  Up V20%  **Dosimetric parameters (absolute)**  Low V5 cc  Up V5 cc  Low V20 cc  Up V20 cc | 0.587  0.593  0.523  0.616  0.507  0.564  0.534  0.609  0.502 | 15.4  28.5  50.6  10.1  31.4  370.9  670.7  191.1  232.7 |  | 4.503  7.136  1.101  8.272  0.483  3.395  1.250  9.936  1.030 | 0.034  0.008  0.294  0.004  0.487  0.065  0.263  0.002  0.310 | |  |

Threshold values represent the optimal cutoff values derived from ROC analysis.

Supplementary Table 4. Multivariate analyses using the Fine-Gray method for Grade ≥ 2 radiation pneumonitis

| **Variables** | **HR** | **95% CI** | ***p*** |
| --- | --- | --- | --- |
| Age | 1.013 | 0.987-1.040 | 0.320 |
| Brinkman Index (< 1060 vs. ≥ 1060) | 1.979 | 1.202-3.260 | 0.007 |
| V5% (< 35.7 vs. ≥ 35.7) | 0.777 | 0.339-1.780 | 0.550 |
| V20% (< 22.5 vs. ≥ 22.5) | 1.905 | 0.698-5.204 | 0.210 |
| Mean lung dose (Gy) (< 15.4 vs. ≥ 15.4) | 1.103 | 0.518-2.350 | 0.800 |
| Low V20 cc (< 191.1 vs. ≥ 191.1) | 2.314 | 1.332-4.020 | 0.003 |

HR, hazard ratio; CI, confidence interval

Supplementary Table 5. cCRT-only analysis (n=86)

| **Parameter** | **ROC analysis** | |  | **Univariate analysis** | | |  |
| --- | --- | --- | --- | --- | --- | --- | --- |
|  | **AUC** | **Threshold** |  | **Statistic (χ^2^)** | | ***p-value*** | |
| **Dosimetric parameters (%)**  V5% | 0.443 | 25.2 |  | 0.057 | 0.812 | |  |
| V20% | 0.625 | 22.7 |  | 6.636 | 0.010 | |  |
| Mean lung dose (Gy)  Low V5%  Up V5%  Low V20%  Up V20%  **Dosimetric parameters (absolute)**  Low V5 cc  Up V5 cc  Low V20 cc  Up V20 cc | 0.622  0.654  0.508  0.675  0.486  0.635  0.548  0.668  0.476 | 14.8  26.4  35.6  17.6  23.9  374.1  670.7  191.1  556.4 |  | 7.241  8.782  1.544  9.984  0.756  8.129  3.379  11.486  0.416 | 0.007  0.003  0.214  0.002  0.385  0.004  0.066  0.001  0.519 | |  |

Threshold values represent the optimal cutoff values derived from ROC analysis.

Supplementary Table 6. Laterality-based analysis (n=145)

| **Parameter** | **ROC analysis** | |  | **Univariate analysis** | | |  |
| --- | --- | --- | --- | --- | --- | --- | --- |
|  | **AUC** | **Threshold** |  | **Statistic (χ^2^)** | | ***p-value*** | |
| **V20%** |  |  |  |  |  | |  |
| Rt upper/middle lobes | 0.600 | ≥ 47.0 |  | 8.533 | 0.003 | |  |
| Rt lower lobe  Lt upper lobe  Lt lower lobe  **V20 cc**  Rt upper/middle lobes  Rt lower lobe  Lt upper lobe  Lt lower lobe | 0.612  0.585  0.569  0.588  0.615  0.579  0.565 | ≥ 33.2  < 21.9  < 0.1  ≥ 454.3  ≥ 174.2  < 200.3  < 0.8 |  | 10.428  5.006  2.353  9.250  11.996  4.032  2.951 | 0.001  0.025  0.125  0.002  0.001  0.045  0.086 | |  |

Thresholds are shown in the direction of higher risk of Grade ≥ 2 radiation pneumonitis.

Threshold values represent the optimal cutoff values derived from ROC analysis.
